# Supplementary material for: Specific binding of Hsp27 and phosphorylated Tau mitigates abnormal Tau aggregation-induced pathology
Source: eLife. 2022 Sep 1;11:e79898. doi: 10.7554/eLife.79898 (PMC9436411; doi:10.7554/eLife.79898)
Supplement: Figure 2—source data 1. [file elife-79898-fig2-data1.doc]

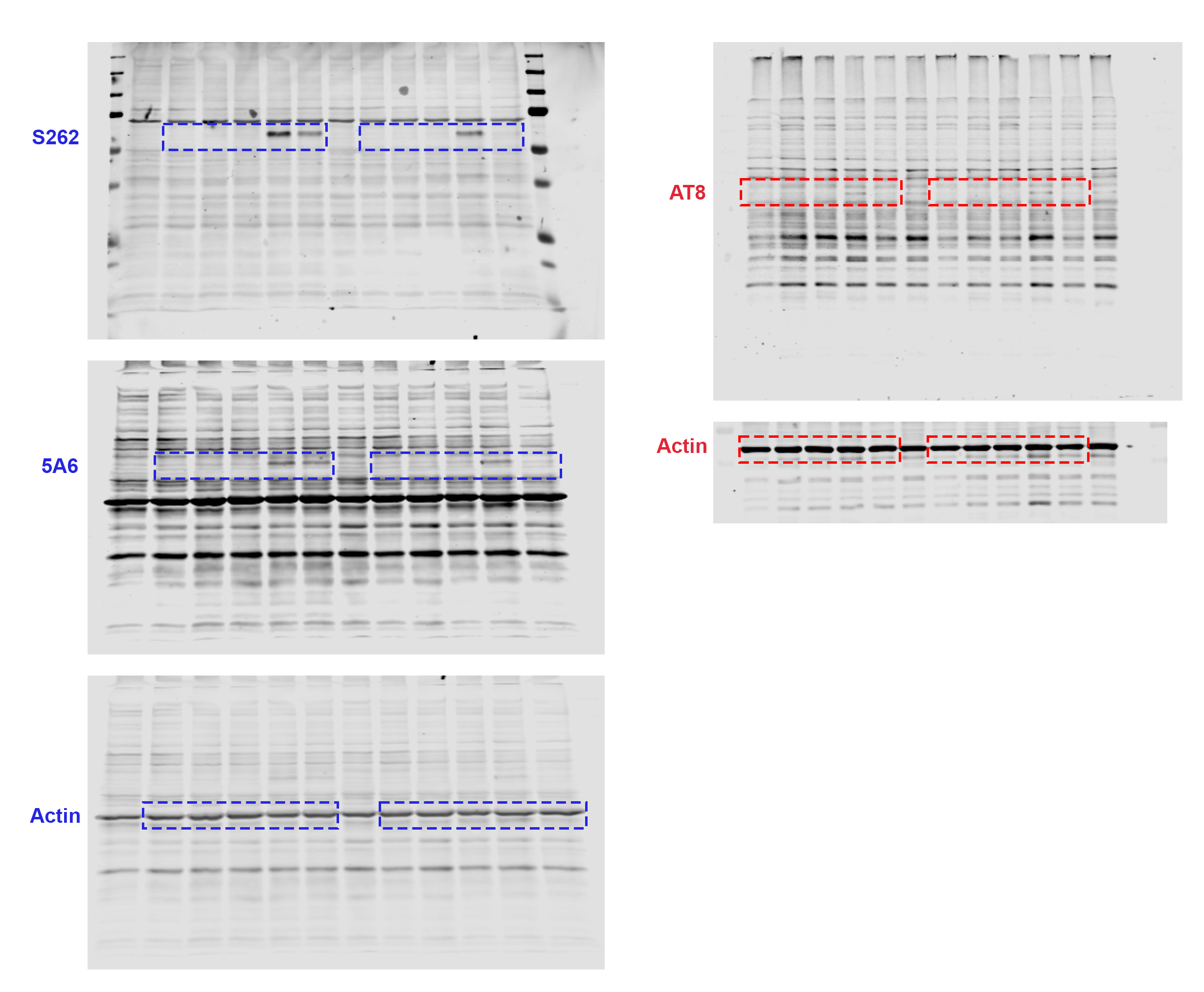


**Figure 2-source data**

The full blots for Figure 2A. Boxed areas highlight the bands included in Figure 2A.
